# Laparoscopic fenestration for a large ruptured splenic cyst combined with an elevated serum carbohydrate antigen 19–9 level: a case report

**DOI:** 10.1186/s12893-019-0517-5

**Published:** 2019-05-30

**Authors:** Yoshitaka Imoto, Takahiro Einama, Makiko Fukumura, Keita Kouzu, Hiromi Nagata, Ayano Matsunaga, Mayumi Hoshikawa, Makoto Nishikawa, Akifumi Kimura, Takuji Noro, Suefumi Aosasa, Hideyuki Shimazaki, Hideki Ueno, Junji Yamamoto

**Affiliations:** 1 Surgery Department, National Defense Medical College, Tokorozawa, Saitama, Japan; 2 Laboratory Department, National Defense Medical College Hospital, Tokorozawa, Saitama, Japan

**Keywords:** Splenic cyst, Elevated CA19–9, Young female, Laparoscopic fenestration, Case report

## Abstract

**Background:**

Non-parasitic splenic cysts are associated with elevated serum carbohydrate antigen (CA) 19–9 levels. We report a case in which a 23-year-old female exhibited a large ruptured splenic cyst and an elevated serum CA19–9 level.

**Case presentation:**

The patient, who experienced postprandial abdominal pain and vomiting, was transferred to our hospital and was found to have a large splenic cyst during an abdominal computed tomography (CT) scan. On physical examination, her vital signs were stable, and she demonstrated rebound tenderness in the epigastric region. An abdominal CT scan revealed abdominal fluid and a low-density region (12 × 12 × 8 cm) with enhanced margins in the spleen. The patient’s serum levels of CA19–9 and CA125 were elevated to 17,580 U/mL and 909 U/mL, respectively. A cytological examination of the ascitic fluid resulted in it being categorized as class II. Finally, we made a diagnosis of a ruptured splenic epidermoid cyst and performed laparoscopic splenic fenestration. The patient’s postoperative course was uneventful, and she was discharged on postoperative day 5. The cystic lesion was histopathologically diagnosed as a true cyst, and the epithelial cells were positive for CA19–9. Follow-up laboratory tests performed at 4 postoperative months showed normal CA19–9 (24.6 U/L) and CA125 (26.8 U/L) levels. No recurrence of the splenic cyst was detected during the 6 months after surgery.

**Conclusion:**

Laparoscopic fenestration of a ruptured splenic cyst was performed to preserve the spleen, after the results of abdominal fluid cytology and MRI were negative for malignancy.

## Background

Non-parasitic splenic cysts can be asymptomatic or present with non-specific symptoms, e.g., pain in the left upper quadrant of the abdomen or increasing abdominal girth [1, 2]. Occasionally, splenic cysts cause complications, such as infections, rupturing, or bleeding. Recent studies have reported that non-parasitic splenic cysts are associated with elevated serum and intracystic levels of carbohydrate antigen 19–9 (CA19–9), CA125, and carcinoembryonic antigen (CEA) [3]. A few cases of malignant cystic splenic tumors that presented as splenic cysts have been reported [4, 5]. It is difficult to distinguish between large benign splenic cysts and malignant lesions in cases involving elevated serum and abdominal fluid levels of CA19–9. Here, we report a case in which a young female presented with a large ruptured splenic cyst together with elevated serum CA19–9 and CA125 levels.

## Case presentation

**Fig. 1:**
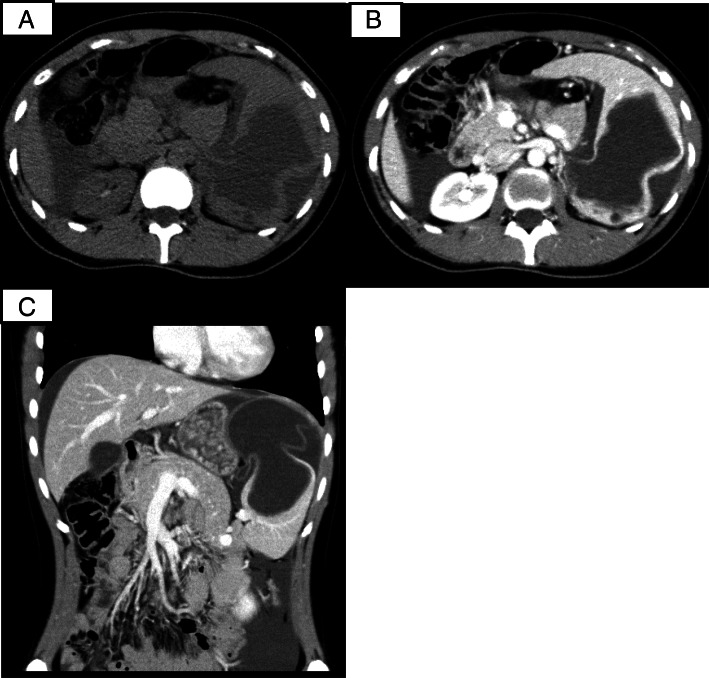
An abdominal CT scan showed a collapsed cystic lesion, measuring 12 cm × 12 cm × 8 cm, in the spleen and abdominal fluid in Morison’s pouch and around the liver and spleen (**a**). Moreover, an 8-mm cyst and a small collapsed cystic lesion were found posterior to the large cystic lesion on a contrast-enhanced CT scan (**b**). There was no evidence of contrast medium extravasation (**c**)

**Fig. 2:**
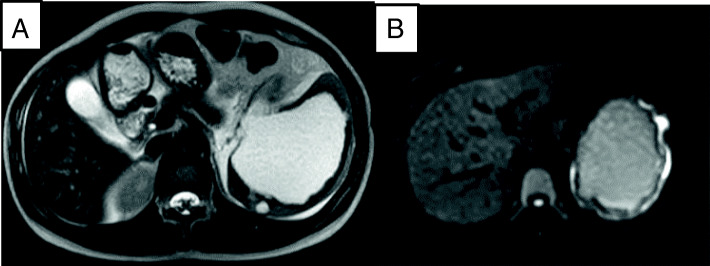
Contrast-enhanced MRI showed a cystic lesion, which exhibited slightly hyperintense signals on the T1- (**a**) and diffusion-weighted sequences and hyperintense signals on the T2-weighted sequence (**b**). No solid components or mural cysts were found in the cyst

A previously healthy 23-year-old female complained of the sudden onset of abdominal pain and vomiting after eating supper and drinking alcohol. She presented to her local hospital’s emergency department. An abdominal computed tomography (CT) scan showed a collapsed cystic lesion and abdominal fluid. A ruptured splenic cyst was suspected, and so the patient was referred to our hospital. On arrival, the patient complained of upper abdominal pain. She stated that she had not suffered any diarrhea, hematemesis, or trauma, nor had she recently come into contact with any sick individuals or gone travelling. She was not taking any regular medication and had no relevant family medical history. She had a slightly elevated temperature (37.3 °C), but the rest of her vital signs were normal. An abdominal examination revealed rebound tenderness in the epigastric region. The initial laboratory tests demonstrated an elevated white blood cell count (18.4 × 10^3^ /L) (predominantly due to increased numbers of neutrophils) and increased serum amylase levels (162 U/L), together with normal hemoglobin and C-reactive protein (CRP) levels. A coagulation screen produced normal results. However, the following tumor marker level measurements were obtained: CA19–9: 17580 U/L (normal: < 37 U/mL), CA125: 909.8 U/L (normal: < 35 U/mL), CEA: 2.5 ng/mL (normal: 5.3 ng/mL), and interleukin-2 receptor (IL-2R): 389 U/L (normal: < 530 U/L). An ascitic tap was obtained, which revealed the following results: lactate dehydrogenase (LDH): 904 U/L, serum total protein (TP): 5.0 g/dL, CA19–9: 490000 U/L, CA125: 24560 U/L, and CEA: 60.6 ng/mL (Table 1). Abdominal fluid cytology revealed no evidence of malignancy. An abdominal CT scan showed a collapsed cystic lesion, measuring 12 × 12 × 8 cm, in the spleen and abdominal fluid in Morison’s pouch and around the liver and spleen. Moreover, an 8-mm cyst and a small collapsed cystic lesion were found posterior to the large cystic splenic lesion. No masses were found in the liver, pancreas, kidneys, or gastrointestinal tract. There was no evidence of contrast medium extravasation (Fig. 1). Based on these results, we excluded a ruptured spleen and made a diagnosis of a ruptured splenic cyst. The differential diagnoses for ruptured epidermoid cysts include splenic pseudocyst, lymphangioma, primary mucinous cystadenocarcinoma, splenic lymphoma, and metastatic tumors. Cystic Echinococcosis were denied because she had denied any history of traveling abroad. There was no evidence of massive hemorrhaging, and an additional contrast-enhanced magnetic resonance imaging (MRI) scan was obtained on the following day. It showed a cystic lesion, which exhibited slightly hyperintense signals on the T1- and diffusion-weighted sequences and hyperintense signals on the T2-weighted sequence. No solid components or mural cysts were found in the cyst (Fig. 2).

**Table 1:** Preoperative laboratory data

Complete blood count		Biochemistry		Abdominal fluid	
WBC	18,400 /μL	AST	27 U/L	Color	Red-brown
Hb	14 g/dL	ALT	7 U/L	CA19–9	490,000 U/mL
Plt	21.4 × 10^4^ /μL	BUN	11 mg/dL	CA125	24,560 U/mL
		Cr	0.71 mg/dL	CEA	60.6 ng/mL
		TP	8.1 g/dL	TP	5 g/dL
		Alb	4.7 g/dL	Alb	3 g/dL
		CRP	< 0.3 mg/dL	LDH	904 U/L
		CA19–9	17,580 U/mL	Specific gravity	1.019
		CA125	909.8 U/mL		
		CEA	2.5 ng/mL	Rivalta test	(+)
		IL-2R	389 U/mL	Cytology	class II
				Culture	negative

*WBC* white blood cells, *Hb* hemoglobin, *Plt* platelets, *AST* aspartate aminotransferase, *ALT* alanine aminotransferase, *BUN* blood urea nitrogen, *Cr* creatinine, *TP* total protein, *Alb* albumin, *CRP* C-reactive protein, *CA* carbohydrate antigen, *CEA* carcinoembryonic antigen, *IL-2R* interleukin-2 receptor, *LDH* lactate dehydrogenase

**Fig. 3:**
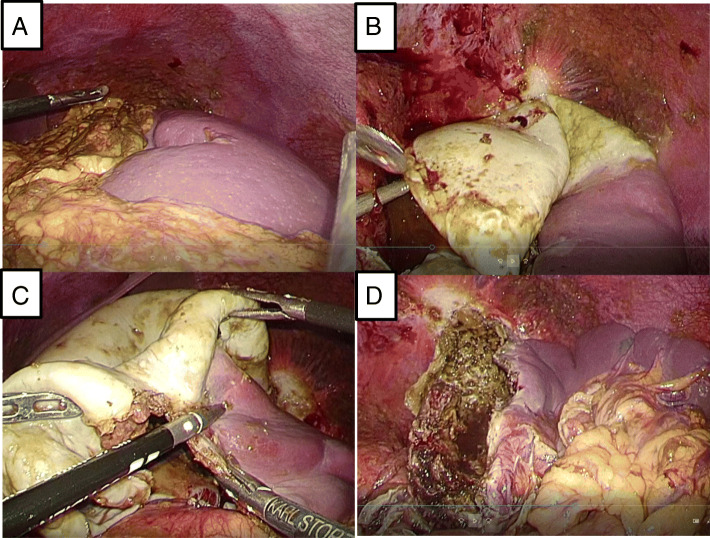
We performed laparoscopic fenestration of the splenic cyst. We dissected the part of the greater omentum that had adhered to the cyst wall (**a**), drained the cystic cavity, and fenestrated the splenic cyst wall using a harmonic scalpel (**b** and **c**). We cauterized the interior of the cyst wall (**d**)

After one week, we removed the splenic cyst via laparoscopic fenestration. Exploration of the surgical field revealed abdominal fluid. The cyst was located at the upper pole of the spleen. We dissected the part of the greater omentum that had adhered to the cyst wall, drained the cyst cavity, and fenestrated the splenic cyst wall using an ultrasonic scalpel, before cauterizing the interior of the cyst wall (Fig. 3). The patient had an uncomplicated postoperative course and was discharged on postoperative day 5.

**Fig. 4:**
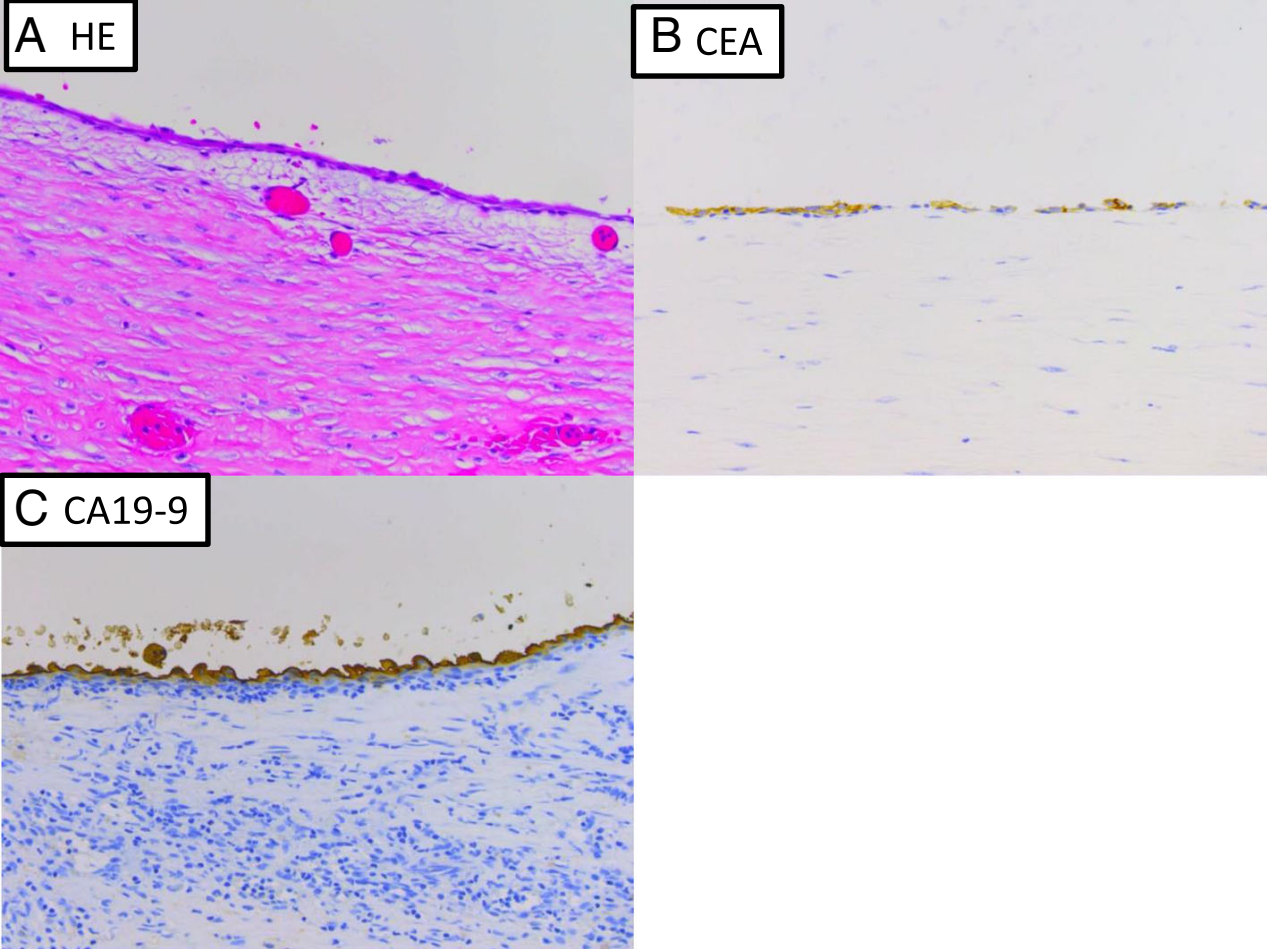
Pathological diagnosis of the resected specimen (Hematoxylin and eosin stain and immunohistochemistry of CA19–9 and CEA). The cyst wall consisted of fibrous tissue and was lined by a single layer or several layers of squamous epithelium (**a**). Immunohistochemistry demonstrated that the epithelial cells were positive for CA19–9 (**b**) and CEA (**c**) (4A, 4B and 4C: × 400)

A pathological examination revealed an epidermoid cyst. The cyst wall consisted of fibrous tissue and was lined by a single layer or several layers of squamous epithelium. Immunohistochemistry demonstrated that the epithelial cells were positive for CA19–9 and CEA (Fig. 4). The patient’s serum levels of CA19–9 and CA125 were 1024 U/mL and 199 U/mL, respectively, at 2 weeks after surgery and had returned to normal at 4 postoperative months (Table 2). A follow-up abdominal CT scan performed at 6 postoperative months did not show any recurrence. The patient was healthy at 15 postoperative months.

**Table 2:** Evaluation of preoperative and postoperative serum tumor marker levels

	Preoperative	16POD	121POD
CA19–9 (U/mL)	17,580	1024	24.6
CA125 (U/mL)	909.8	198.9	26.8
CEA (ng/mL)	2.5	1.7	2.1

*POD* postoperative day

## Discussion and conclusion

The preoperative diagnosis was a ruptured non-parasitic splenic cyst. Cystic Echinococcosis were ruled out by her social history. In Japan, cystic Echinococcosis is very rare disorders, except for imported cases [6] and hydatid serological testing may be indicated in patients who have been to an endemic area of Echinococcosis. Major problem is to differentiate epidermoid cysts from malignant lesions in cases involving elevated serum levels of tumor markers. The sensitivity of abdominal fluid cytology for malignant ascites is about 60 to 70% with 90 to 100% specificity and 11.7% (26/222) of negative cytology cases have a malignant tumor [7].

To the best of our knowledge, 51 cases of splenic cysts involving high levels of tumor markers have been reported, not including accessory spleens [1, 3, 8–41] (Table 3). Of 50 cases, 76% were female (38 cases). The mean age was 28 years old (range: 9–62). No parasitic splenic cysts cases involving elevated tumor markers were found. Including our case, only 4 cases of ruptured splenic cysts have been reported [15, 24, 28]. All of these cases involved abdominal pain. One case involved traumatic rupture [24]. Only one of the patients with ruptured splenic cysts developed peritonitis, which required emergency laparotomy, and purulent ascites was detected, but the route by which splenic cysts become infected is unknown [28]. Among the 51 cases, the mean size of the ruptured cysts was 12.6 cm, while that of the unruptured cysts was 12.7 cm (range: 3–25). The cases involving ruptured cysts demonstrated higher serum CA19–9 levels than those involving unruptured cysts (21,199 U/L vs. 2907 U/L, respectively). However, there was no correlation between cyst size and the serum CA19–9 level (r = − 0.057). The postoperative serum levels of tumor markers were not mentioned in 4 cases, whereas they decreased after treatment in 47 cases. The tumor marker levels of 38 of the 47 cases subsequently normalized. These findings suggest that measuring the serum CA19–9 level is useful for evaluating the effects of treatments for splenic epidermoid cysts. Four patients experienced recurrence after the following treatments: surgery (2 cases) [27, 37], percutaneous drainage alone (one case) [11], and percutaneous drainage and sclerotherapy (one case) [9]. The serum CA19–9 levels before and after recurrence were not mentioned in 4 cases, so it is unclear whether measuring the serum CA19–9 level is useful for evaluating recurrence.

**Table 3:** Cases of splenic cysts presenting with elevated serum tumor marker levels

Factor			51
Male/female			12/38
Not described			1
Age (mean ± SD)			28 ± 11
Size (mean ± SD)			12.7 ± 4.7
Chief complaint	Abdominal pain		23
	Discomfort, fullness, or heavy feeling		10
	Fever		5
	Back pain		4
	Shoulder pain		2
	None		7
	Others		5
	Not described		10
State of cyst	No complications		44
	Complicated		7
		Rupture	4
		Infection	4
		Hematoma	1
Tumor markers	CA19–9		50
(high serum levels)	CA125		7
	CEA		8
	Others		3
Surgical procedure	Splenectomy (laparoscopic)		30 (8)
	Partial splenectomy (laparoscopic)		1 (0)
	Cystectomy (laparoscopic)		4 (2)
	Fenestration (laparoscopic)		4 (4)
	Percutaneous aspiration		14
	Not described		1
Pathology	Epithelial cyst		35
	Mesothelial cyst		2
	Pseudocyst		1
	Not described		14

*SD* standard deviation

The mechanism responsible for the elevated tumor marker levels seen in such cases remains unclear. The intracystic and serum CA19–9 levels were high in most cases, but the CEA levels were markedly elevated in the cystic fluid whereas normal in the serum in some cases [27, 28, 32]. The inner epithelial cells of splenic cysts were immunohistochemically positive for CA19–9 in most cases and CEA in some cases [15, 18], so they can produce tumor markers and over time these markers become concentrated in the cysts, which are closed cavities. Tumor markers are secreted into the bloodstream via an unknown mechanism. If a cyst ruptures, the serum levels of tumor markers increase because the cystic fluid containing the markers is absorbed through the peritoneum.

Large, symptomatic, or complex cysts are indicated for treatment. Large cysts are susceptible to rupture and can cause other complications. According to some previous reports, cysts measuring ≥5 cm in diameter are indicated for surgical intervention, but there is a lack of evidence for this 5-cm cut-off point, and cyst size is generally not used as a cause for intervention [42].

The treatments for ruptured splenic cysts include percutaneous drainage, splenectomy, partial splenectomy, marsupialization, and fenestration. Percutaneous aspiration therapy alone is associated with low success and high recurrence rates [11]. Percutaneous aspiration combined with the injection of sclerosing substances (e.g., tetracycline, minocycline, or ethanol) is used to prevent recurrence. Percutaneous ethanol ablation is associated with high success rates, but the associated recurrence rates vary [39, 43]. Surgical intervention is recommended in symptomatic or complicated cases [44]. Splenectomy can prevent recurrence, but carries a risk of postoperative infection and thrombocytosis. Epidermoid cysts predominantly occur in young females. Thus, spleen-preserving surgery (such as laparoscopic fenestration, marsupialization, or dome resection) has been suggested, especially for cysts located at the poles of the spleen. The resection of the cyst wall and the cauterization of the rest of the cyst wall are recommended to avoid recurrence [11]. Splenectomy was performed in all 3 of the previously reported cases of ruptured cysts with elevated serum CA19–9 levels. In the present case, laparoscopic fenestration of the splenic cyst, rather than laparoscopic splenectomy, was selected because the results of cytology and MRI are negative for malignancy.

## Abbreviations

CA: carbohydrate antigen
CEA: carcinoembryonic antigen
CRP: C-reactive protein
CT: computed tomography
IL-2R: interleukin-2 receptor
LDH: lactate dehydrogenase
MRI: magnetic resonance imaging
TP: total protein
